# Effect of diets supplemented with coated plant essential oil on the growth performance, immunity, antioxidant activity, and fecal microbiota of weaned piglets

**DOI:** 10.3389/fvets.2024.1346922

**Published:** 2024-03-11

**Authors:** Yu Niu, Yiying Chen, Jinsong Liu, Yulan Liu, Shiping Xiao, Caimei Yang, Ting Yang, Weiwei Huan

**Affiliations:** ^1^College of Animal Science and Technology and College of Veterinary Medicine, Zhejiang A & F University, Hangzhou, Zhejiang, China; ^2^Zhejiang Huijia Biotechnology Co., Ltd., Huzhou, Zhejiang, China; ^3^College of Chemistry and Materials Engineering, Zhejiang A & F University, Hangzhou, Zhejiang, China

**Keywords:** plant essential oil, weaned piglet, immunity, antioxidant, microbiota

## Abstract

**Introduction:**

This trial was conducted to compare the effect of diets supplemented with plant essential oil (PEO) and coated plant essential oil (CEO) on growth performance, immunity, antioxidant activity, and fecal microbiota of weaned piglets.

**Methods:**

A total of 360 21-day-old weaned piglets were randomly allocated into three groups, namely, CON, PEO, and CEO (basal diets supplemented with 0, 500 mg/kg PEO, and 500 mg/kg CEO, respectively) for a 4-week feeding trial.

**Results and discussion:**

The results showed that dietary supplementation with CEO improved the average final weight and average daily gain, decreased the diarrhea rate, increased antioxidant enzyme activities, enhanced immunoglobulin concentrations, and decreased concentrations of pro-inflammatory cytokines in the serum of weaned piglets (*p* < 0.05). In addition, CEO addition increased the fecal concentrations of propionic acid and isovaleric acid of piglets (*p* < 0.05). Spearman correlation analysis showed that fecal microorganisms at the genus level were closely correlated with the volatile fatty acid concentrations. The present study indicated that PEO and CEO could improve growth performance, enhance immunity, and increase antioxidant capacity by modulating the microbial flora in weaned piglets. Moreover, CEO addition seemed to offer more positive results than of PEO addition.

## 1 Introduction

Piglets are exposed to various stressors, including weaning, oxidative stress, pathogenic bacteria, and inflammation, resulting in declining growth performance and high mortality and morbidity (1). Recently, phytogenetic feed additives as a potential alternative to antibiotics have attracted great interest in the swine industry. Plant essential oil (PEO) is a kind of volatile aromatic substance extracted from some aromatic plants, such as *Cinnamomum cassia*, *Thymus mongolicus,* and *Vanilla planifolia* (2). PEO is mainly composed of terpenoids, ketones, alcohols, and aldehydes and exerts antibacterial, antioxidant, and anti-inflammatory properties (3, 4). Due to the excellent biological function of PEO, it has become one of the recognized products used to replace antibiotics in pigs (5, 6). Research has shown that PEO, consisting of eucalyptus essential oil (3%), oregano essential oil (2%), thyme essential oil (0.5%), lemon essential oil (0.1%), garlic essential oil (0.1%), and coconut oil (5%), increased growth performance and nutrient digestibility and improved pork quality by increasing the anti-oxidant capacity of finishing pigs (7). Studies have also demonstrated that PEO supplementation (13.5% thymol and 4.5% cinnamyl aldehyde) improved growth performance, immune function, antioxidant activities, and intestinal health in weaned pigs (8, 9). Hall et al. (10) found that oregano essential oil fed to matern could improve the growth performance and intestinal health of piglets by regulating gut microbiota. Therefore, previous studies have suggested that PEO supplementation may improve pig performance, antioxidant activity, immunity, and intestinal microecology (1, 11).

However, the application of plant essential oil in feed additives is limited because of its characteristics, such as easy volatilization, high volatility and reactivity, and poor water solubility (12). At present, PEO microcapsules mainly use a fat-coating process to improve the oxidative stability, thermal stability, and biological activity of the core material (13). Although microencapsulation can make PEO pass through the stomach, it is difficult to release and function in the cecum and colon due to its insolubility in water. Therefore, after years of observation and research, we have found that the combination of water dispersion technology and cross-linking coated technology may be the development direction of PEO preparation technology (14). Water dispersion technology means the emulsification and homogenization of PEO and water dispersion materials, which can achieve the water solubility of essential oil. The cross-linking coating process refers to the use of amino groups in polymer materials to react with the carbonyl groups of sugars under appropriate temperature and pressure conditions to form a crisscrossing grid structure, finally embedding the core material inside and forming a hardened layer on the surface of the particles. Through water dispersion and the cross-linking coating process, PEO is allowed to pass through the stomach and is sustainably released in the intestine so that PEO can fully exert antibacterial and antioxidant effects in the intestine (15).

However, studies that investigate the effect of coated plant essential oil (CEO) on the growth performance, antioxidant activity, and immunity of weaned piglets are limited. Therefore, the aim of this study was to compare the effect of PEO and its cross-linked coating form on weaned piglets and explore whether coated technology could improve the bioavailability of compound essential oil consisting of cinnamaldehyde, thymol, and vanillin. This study will provide a reference for the development and efficient utilization of CEO in weaned piglets.

## 2 Materials and methods

### 2.1 Preparation of plant essential oil

PEO and CEO in this study were provided by Zhejiang Huijia Biotechnology Co., Ltd. (Anji, Zhejiang, China), which consisted of 27% cinnamaldehyde, 9% thymol, and 4% vanillin, respectively.

The specific preparation method of CEO was to first emulsify PEO with emulsifier and then add the fat with a high solubility point. After being mixed evenly, the small molecule dextrin, denatured starch, and water-based enteric resin were added to the mixture, which was then homogenized to the emulsion with the homogenizer and formed a microsphere with network structure through pressure spraying. As the microsphere fell, they were coated with starch and then dried (16).

### 2.2 Experimental design and animal management

The experiment was conducted at Chia Tai Pig Industry (Yuyao) Co., Ltd. (Yuyao, Zhejiang, China). After weaning at 21 days of age, 360 commercial crossbred (Duroc ×Yorkshire × Landrace) weaned piglets with average initial weight (8.02 ± 0.45) kg were randomly divided into three groups with six replications of 20 piglets per replication for 4 weeks. The three treatment groups were allocated to the control group (CON, basal diet), PEO group (basal diet containing 500 mg/kg PEO), and CEO group (basal diet containing 500 mg/kg CEO). The basal diets were formulated to meet the nutritional requirements of piglets according to National Research Council 2012 requirements. The ingredients and nutrient composition of the basal diet are shown in Table 1. Environmental pens (3.5 m × 3.5 m) housed 15 piglets each. The house temperature was thermostatically controlled at 25 to 28°C, and the humidity was maintained at 50–60%. All piglets had *ad libitum* access to feed and water during the 4-week feeding trial. This experiment was conducted under the supervision of the Animal Ethics Committee of Zhejiang A & F University following the requirements for the protection and use of laboratory animals (ZAFUAC2022005).

**Table 1:** Composition and nutrient levels of basal diet (air-dried basis).

Item	Content (%)	Item	Content (%)
Ingredient		Nutrient level^2^	
Corn	65.00	Digestive energy (MJ/kg)	14.58
Soybean meal, 46%	10.00	Crude protein	18.15
Fish meal	4.00	Lysine	1.30
Extruded soybean	8.00	Methionine	0.32
Whey powder	5.00	Methionine + Cystine	0.60
Fermented soybean meal	4.00	Threonine	0.83
Premix^1^	4.00	Calcium	0.71
Total	100.00	Total phosphorus	0.72
		Available phosphorus	0.27

^1^The premix provided per kilogram diet: retinyl palmitate, 4.79 mg; cholecalciferol, 0.075 mg; α-tocopherol acetate, 100 mg; menadione, 3 mg; thiamine, 3 mg; riboflavin, 8 mg; nicotinamide, 5 mg; cobalamin, 0.04 mg; biotin, 0.3 mg; pantothenic acid, 20 mg; niacin, 45 mg; folic acid, 2 mg; choline chloride, 450 mg; Fe (from ferrous sulfate), 180 mg; Cu (from copper sulfate), 230 mg; Zn (from zinc oxide), 65 mg; Mn (from manganese sulfate), 50 mg; I (from potassium iodate), 0.5 mg; Se (from sodium selenite), 0.2 mg.

^2^All nutrient levels were calculated values.

### 2.3 Sample collection

On day 49 of age, six piglets with nearly average body weight (one piglet per replicate) from each group were selected and weighed. Approximately 5 mL blood samples were collected in the sterile tube by jugular venipuncture and stayed for 1 h at room temperature for clotting. The blood samples were centrifugated at 3,000 × g at 4°C for 15 min to obtain serum and then stored at −20°C for antioxidant and immune indexes analysis. Fresh feces of six piglets from each group were aseptically collected in the sterile tube by rectal stimulation on day 49 and stored at −80°C for volatile fatty acids (VFA) analysis and microbiota composition.

### 2.4 Growth performance and diarrhea rate

On day 21 and day 49 of age, the body weight of individual piglets was measured. Supplied and leftover feed per pen were recorded daily during the whole experiment. Average daily gain (ADG), average daily feed intake (ADFI), and feed-to-gain ratio (F/G) were calculated for each pen.

The fecal quality of piglets was scored every morning by two independent observers from day 21 to day 49 using a 4-scale scoring method (0 = normally shaped feces, 1 = soft feces, 2 = mildly fluid feces, and 3 = severely watery and frothy feces) according to a previous study (17). Piglets with an average fecal score of >1 were recorded as having diarrhea. The diarrhea rate was calculated according to the following formula:

Diarrhea rate (%) = [the number of diarrhea pigs × diarrhea days / (total number of pigs × experimental days)] × 100

### 2.5 The antioxidant activity analysis in serum samples

The activities of total antioxidant capacity (T-AOC), total superoxide dismutase (T-SOD), glutathione peroxidase (GSH-Px), catalase (CAT), and the levels of malondialdehyde (MDA) concentrations in the serum were determined using corresponding diagnostic kits of Nanjing Jiancheng Bioengineering Institute (Nanjing, Jiangsu, China) according to the instructions of the manufacturer.

### 2.6 The immune function analysis in serum samples

The concentrations of interleukin 1β (IL-1β), interleukin 6 (IL-6), tumor necrosis factor α (TNF-α), immunoglobulin A (IgA), and immunoglobulin G (IgG), immunoglobulin M (IgM) in the serum were determined by Enzyme-linked immunosorbent assay (ELISA) corresponding diagnostic kits (AngleGene, China) according to the manufacturer’s instructions (Nanjing Aoqing Biotechnology Co., Ltd., Nanjing, China).

### 2.7 Volatile fatty acid analysis in fecal sample

Approximately 2 g of fecal sample was added with pre-cooled ultrapure water at a ratio of 1:1 (wt:vol) and centrifuged at 12,000 × g for 10 min at 4°C. The supernatant was obtained and mixed with 25% phosphorous acid (1,5, vol:vol). Then the supernatant was centrifuged at 12,000 × g for 10 min at 4°C and filtered through a membrane filter (pore size 0.45 μm). The VFA concentration of feces was determined using GC7890B gas chromatography with a column (122–3232, DBFFAP, 30 m × 0.25 mm × 0.25 mm, Agilent Technologies, USA).

### 2.8 Analysis of microbial diversity in fecal sample

Microbial DNA was extracted from fecal samples using an MP-soil kit according to the manufacturer’s protocols (Omega Bio-tek, Norcross, GA, US). The final DNA concentration and purification were determined by NanoDrop 2000 UV–vis spectrophotometer (Thermo Scientific, Wilmington, USA), and DNA quality was checked by 1% agarose gel electrophoresis. The V3-V4 hypervariable regions of the bacteria 16S rRNA gene were amplified with primers 338F (5’-ACTCCTACGGGAGGCAGCAG-3′) and 806R (5’-GGACTACHVGGGTWTCTAAT-3′) by thermocycler PCR system (GeneAmp 9,700, ABI, USA) based on Illumina MiSeq platform (Illumina, San Diego, USA) according to the standard protocols by Majorbio Bio-Pharm Technology Co. Ltd. (Shanghai, China). Amplicon sequence variants (ASV) were clustered with a 97% similarity cutoff using UPARSE (version 7.1).^1^ The analysis of alpha diversity (Shannon index, Simpson index, Ace index, Chao index, and Sobs index), beta diversity (PCoA analysis), and microbial composition were performed based on the ASV level. The correlation between the fecal microbiota and VFA concentrations was determined by Spearman analysis.

### 2.9 Statistical analysis

All data were analyzed by one-way analysis of variance (ANOVA) using SPSS statistical software (Ver. 20.0 for Windows, SPSS, Inc., Chicago, IL, USA). The statistical differences in growth performance, diarrhea rate, immune indices, antioxidant activity, and VFA concentrations between treatments were determined by a Ducan’s test. Statistical differences in alpha diversity and microbial community species among different groups were measured using the Kruskal-Wallis H test. The figures were made by GraphPad Prism software (Ver 6.0, San Diego, CA). The probability level of *p* < 0.05 was considered significant difference.

## 3 Results

### 3.1 Growth performance and diarrhea rate

The effect of plant essential oil on the growth performance and diarrhea rate of weaned piglets is shown in Table 2. The results showed that diets supplemented with PEO and CEO improved (*p* < 0.05) average final weight and ADG, decreased feed-to-gain ratio and reduced the diarrhea rate of weaned piglets. The average final weight and ADG in the CEO group were significantly higher than those of the PEO group (*p* < 0.05).

**Table 2:** The effect of plant essential oil on growth performance of weaned piglets.

Item^1^	Treatment groups^2^			*p* Value
	CON	PEO	CEO	
Average initial weight (kg)	8.08 ± 0.03	7.91 ± 0.05	8.07 ± 0.09	0.100
Average final weight (kg)	14.11 ± 0.15^c^	15.60 ± 0.33^b^	16.91 ± 0.30^a^	<0.001
ADG (g)	215.43 ± 5.82^c^	274.7 ± 12.38^b^	315.87 ± 12.15^a^	<0.001
ADFI (g)	400.19 ± 9.35	446.92 ± 35.04	447.64 ± 13.76	0.260
F/G	1.86 ± 0.02^a^	1.63 ± 0.10^b^	1.42 ± 0.05^b^	0.002
Diarrhea rate (%)	4.60 ± 0.31^a^	2.54 ± 0.47^b^	1.75 ± 0.39^b^	<0.001

^a–c^Means within a row with different superscripts differ significantly (*p* < 0.05). Data are presented as mean ± SEM (*n* = 6). ^1^ADG, average daily gain; ADFI, average daily feed intake; F/G, feed to gain ratio. ^2^CON, control group, piglets received basal diet; PEO, piglets received basal diets supplemented with 500 mg/kg plant essential oil (PEO); CEO, piglets received basal diets supplemented with 500 mg/kg cross-linked coated plant essential oil (CEO).

### 3.2 Antioxidant activity

**Figure 1:**
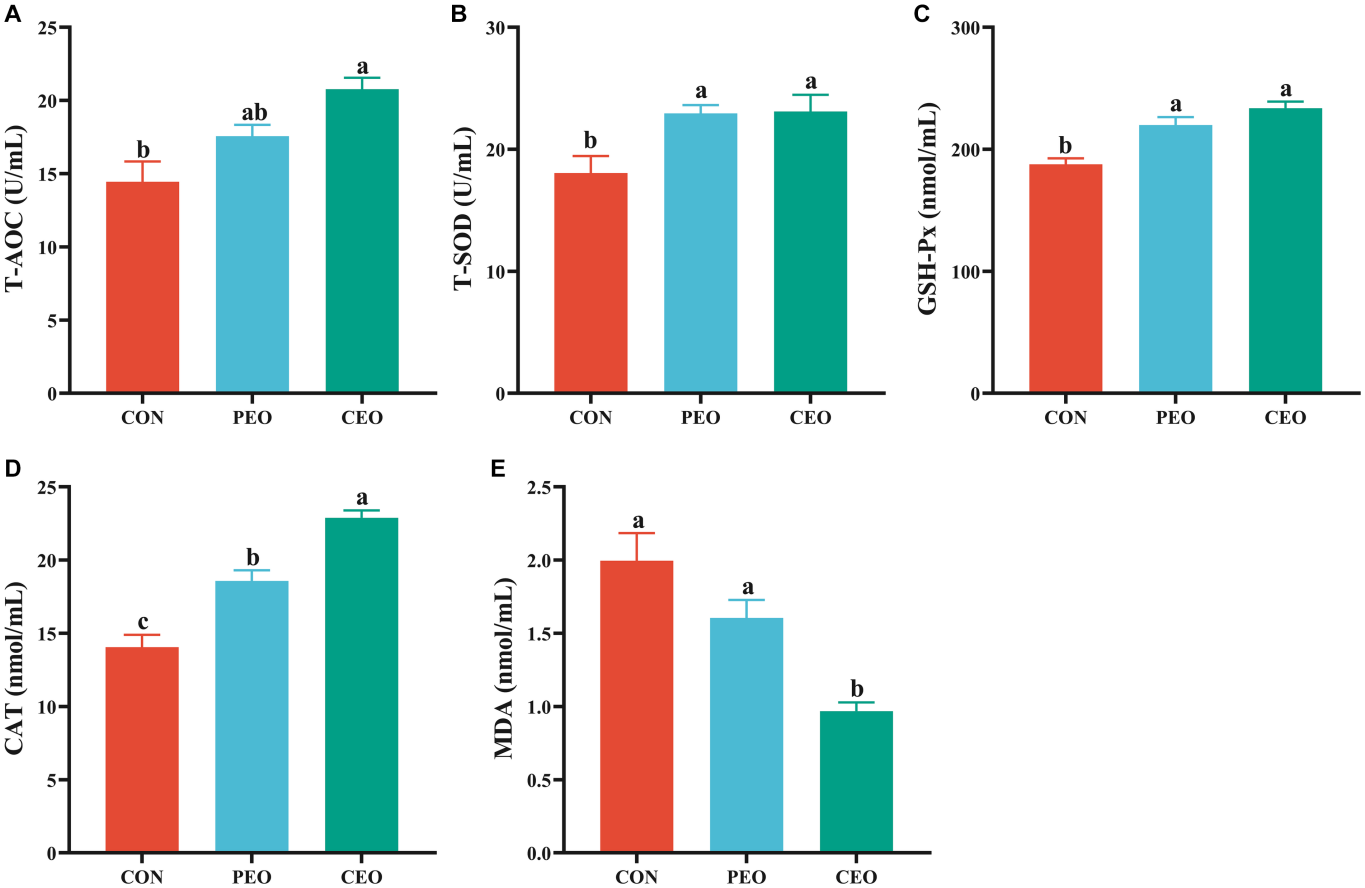
The effect of plant essential oil on the activities of T-AOC **(A)**, T-SOD **(B)**, GSH-Px **(C)**, CAT **(D)** and MDA concentration **(E)** in the serum of weaned piglets. CON, control group, piglets received bas al diet; PEO, piglets received basal diets supplemented with 500 mg/kg plant essential oil (PEO); CEO, piglets received basal diets supplemented with 500 mg/kg coated plant essential oil (CEO). Data are presented as mean ± SEM (*n* = 6). ^a-c^ Means within columns with different superscripts differ significantly (*p* < 0.05). T-AOC, total antioxidant capacity; T-SOD, total superoxide dismutase; GSH-Px, glutathione peroxidase; CAT, catalase; MDA, malondialdehyde.

As shown in Figure 1, dietary supplementation with PEO and CEO increased (*p* < 0.05) the activities of T-SOD, GSH-Px, and CAT of weaned piglets. The level of T-AOC was increased (*p* < 0.05) and MDA concentration was decreased (*p* < 0.05) in the CEO group compared with the CON group. Diets supplemented with CEO increased CAT activity and decreased MDA concentration in the serum of weaned piglets compared with the PEO group (*p* < 0.05).

### 3.3 Immune function

**Figure 2:**
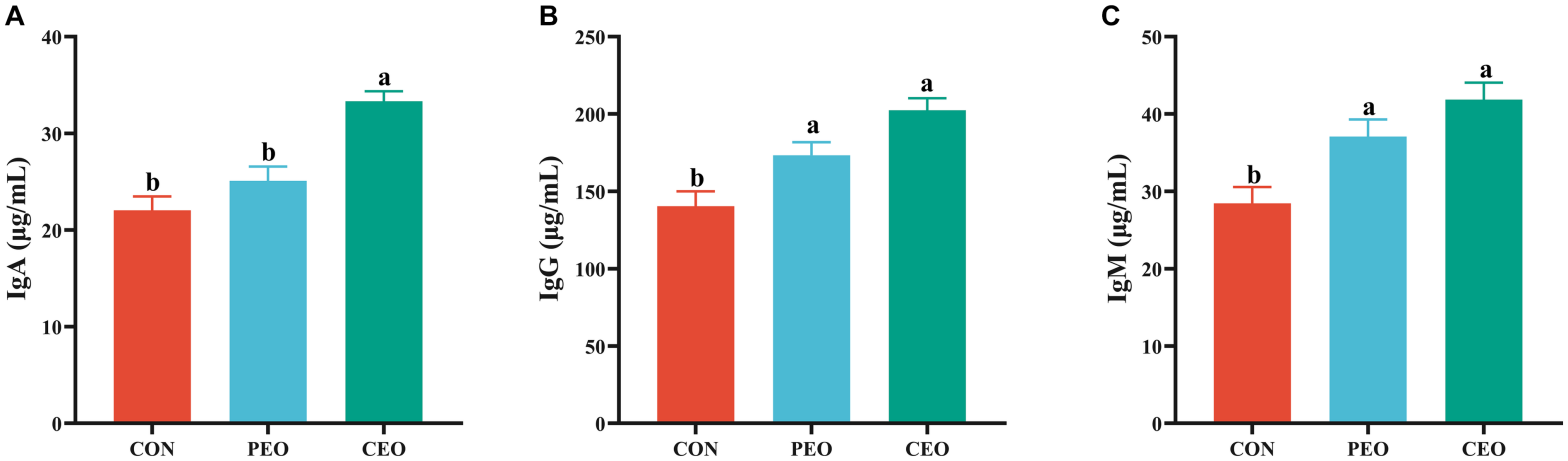
The effect of plant essential oil on the concentrations of IgA **(A)**, IgG **(B)** and IgM **(C)** in the serum of weaned piglets. CON, control group, piglets received basal diet; PEO, piglets received basal diets supplemented with 500 mg/kg plant essential oil (PEO); CEO, piglets received basal diets supplemented with 500 mg/kg coated plant essential oil (CEO). Data are presented as mean ± SEM (*n* = 6). ^a, b^ Means within columns with different superscripts differ significantly (*p* < 0.05). IgA, immunoglobulin A; IgG, immunoglobulin G; IgM, immunoglobulin M.

**Figure 3:**
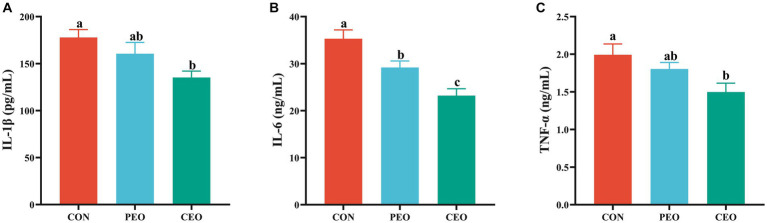
The effect of plant essential oil on the concentrations of IL-1β **(A)**, IL-6 **(B)** and TNF-α **(C)** in the serum of weaned piglets. CON, control group, piglets received basal diet; PEO, piglets received basal diets supplemented with 500 mg/kg plant essential oil (PEO); CEO, piglets received basal diets supplemented with 500 mg/kg coated plant essential oil (CEO). Data are presented as mean ± SEM (*n* = 6). ^a-c^ Means within columns with different superscripts differ significantly (*p* < 0.05). IL-1β, interleukin 1β; IL-6, interleukin 6; TNF-α, tumor necrosis factor α.

The effect of plant essential oil on immunoglobulin content in the serum of weaned piglets is presented in Figure 2. The IgA level in the serum of the CEO group was higher (*p* < 0.05) than in the CON group. Diets supplemented with PEO and CEO enhanced (*p* < 0.05) the concentrations of IgG and IgM in the serum of weaned piglets. The effect of plant essential oil on the inflammatory factors in the serum of weaned piglets is presented in Figure 3. Supplementation of CEO decreased (*p* < 0.05) serum concentrations of IL-1β and TNF-α in weaned piglets. Both PEO and CEO inclusion decreased (*p* < 0.05) IL-6 levels in the serum of weaned piglets. The concentration of IgA was elevated and IL-6 content was lower in the CEO group than in the PEO group (*p* < 0.05).

### 3.4 Volatile fatty acids

**Figure 4:**
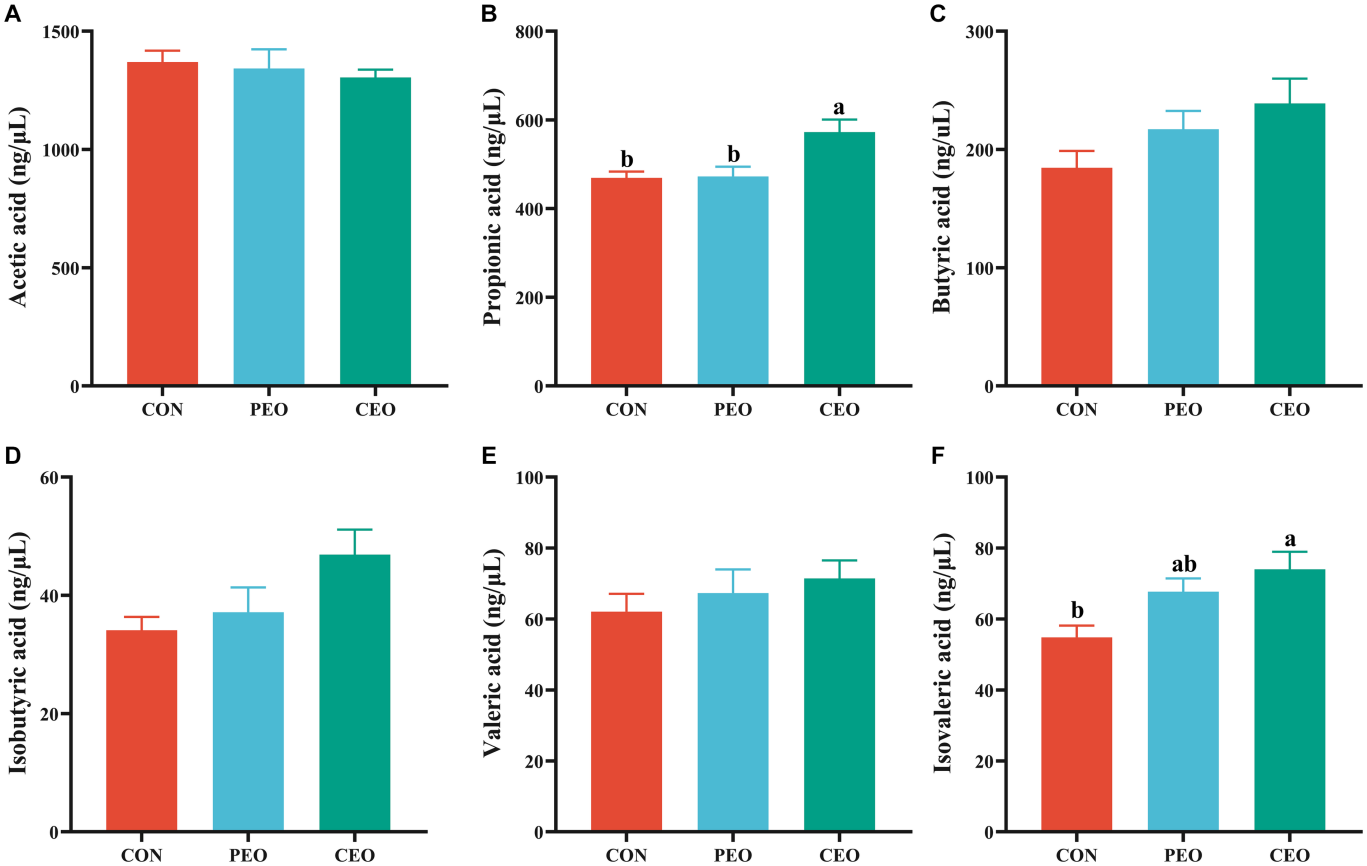
The effect of plant essential oil on the concentrations of acetic acid **(A)**, propionic acid **(B)**, butyric acid **(C)**, isobutyric acid **(D)**, valeric acid **(E)** and isovaleric acid **(F)** in the feces of weaned piglets. CON, control group, piglets received basal diet; PEO, piglets received basal diets supplemented with 500 mg/kg plant essential oil (PEO); CEO, piglets received basal diets supplemented with 500 mg/kg coated plant essential oil (CEO). Data are presented as mean ± SEM (*n* = 6). ^a, b^ Means within columns with different superscripts differ significantly (*p* < 0.05).

As presented in Figure 4, diets supplemented with CEO increased (*p* < 0.05) the concentrations of propionic acid and isovaleric acid in the feces of weaned piglets. No significant differences in the levels of acetic acid, butyric acid, isobutyric acid, and valeric acid were observed between the three groups (*p* > 0.05).

### 3.5 Microbiota composition

**Figure 5:**
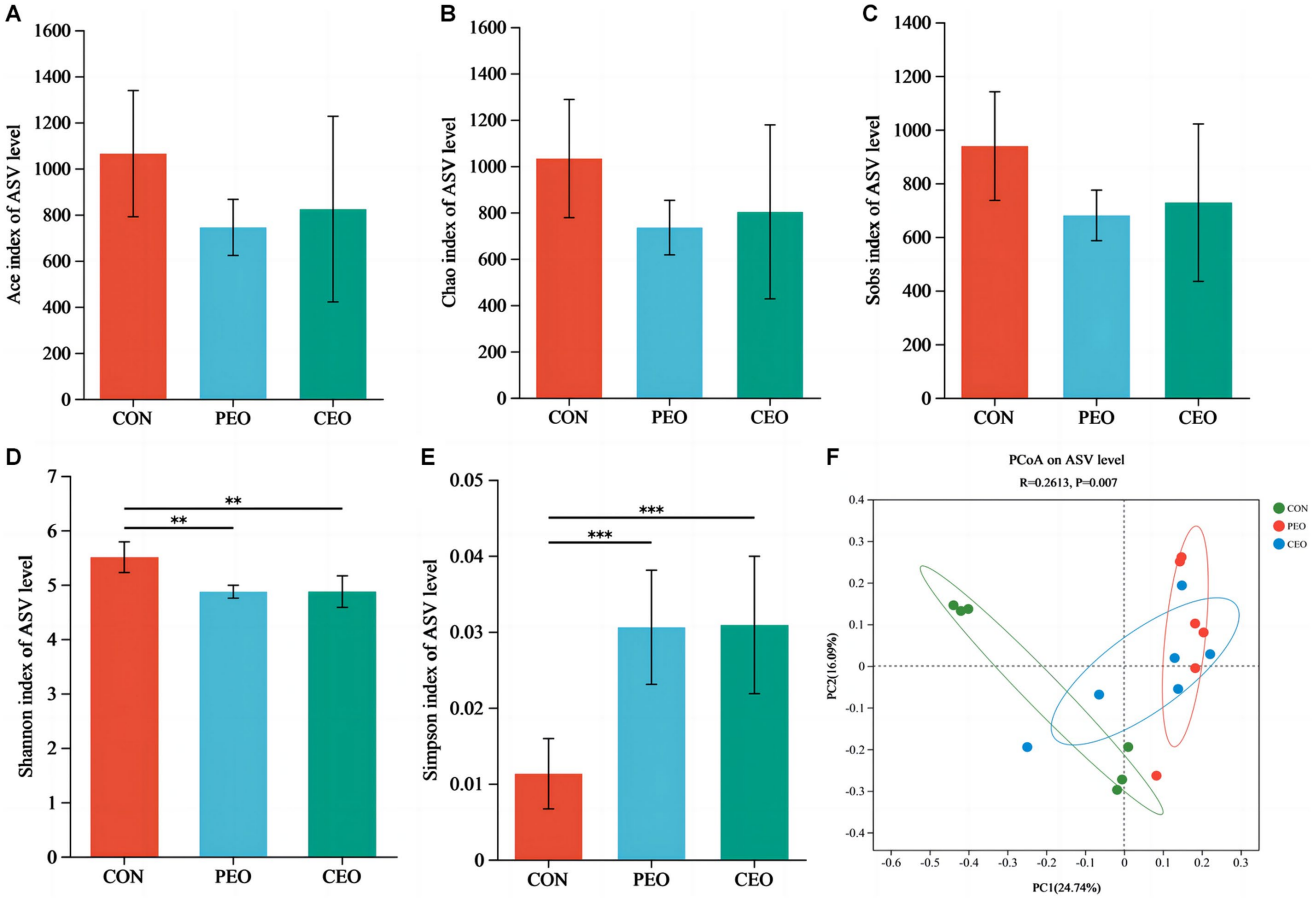
The effect of plant essential oil on alpha diversity **(A–E)** and beta diversity **(F)** in the feces of weaned piglets. CON, control group, piglets received basal diet; PEO, piglets received basal diets supplemented with 500 mg/kg plant essential oil (PEO); CEO, piglets received basal diets supplemented with 500 mg/kg coated plant essential oil (CEO). ^**^p < 0.01; ^***^p < 0.001.

**Figure 6:**
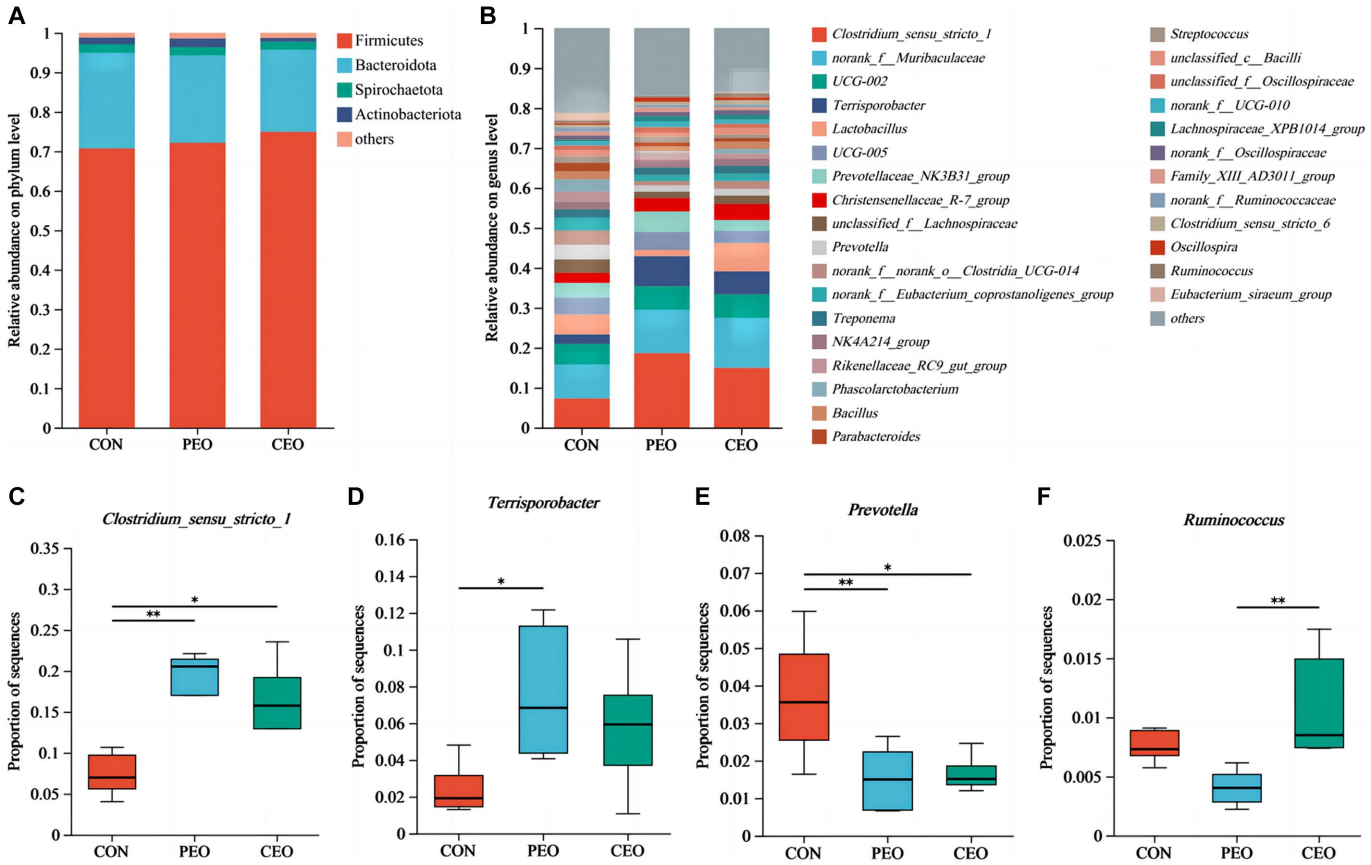
The effect of plant essential oil on the relative abundance of the microbial community at the phylum level **(A)** and genus level **(B–F)** in the feces of weaned piglets. CON, control group, piglets received basal diet; PEO, piglets received basal diets supplemented with 500 mg/kg plant essential oil (PEO); CEO, piglets received basal diets supplemented with 500 mg/kg coated plant essential oil (CEO). ^**^p < 0.01; ^***^p < 0.001.

**Figure 7:**
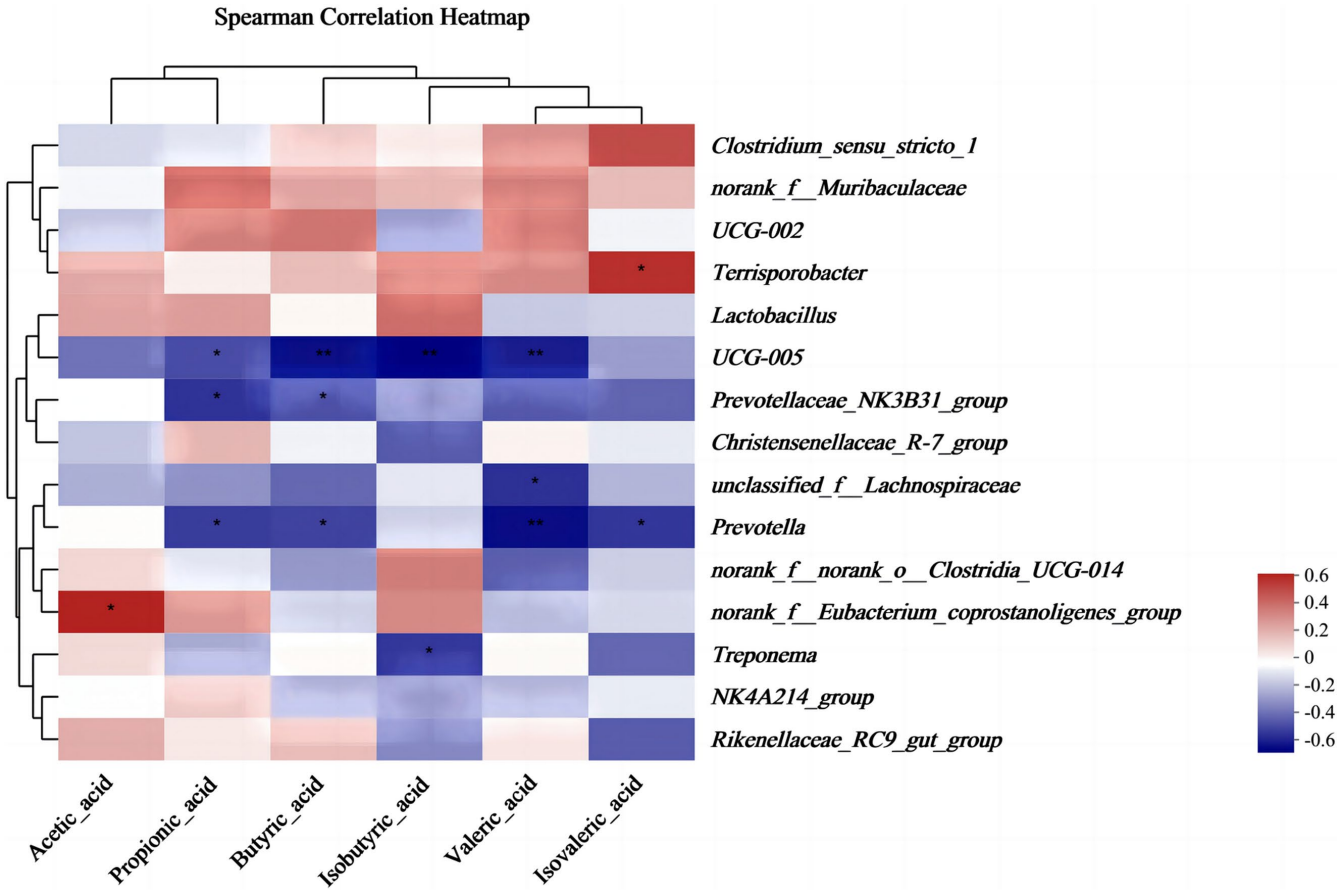
The effect of plant essential oil on the correlation between microbiota and volatile fatty acids in the feces of weaned piglets. CON, control group, piglets received basal diet; PEO, piglets received basal diets supplemented with 500 mg/kg plant essential oil (PEO); CEO, piglets received basal diets supplemented with 500 mg/kg coated plant essential oil (CEO). ^**^p < 0.01; ^***^p < 0.001.

**Figure 8:**
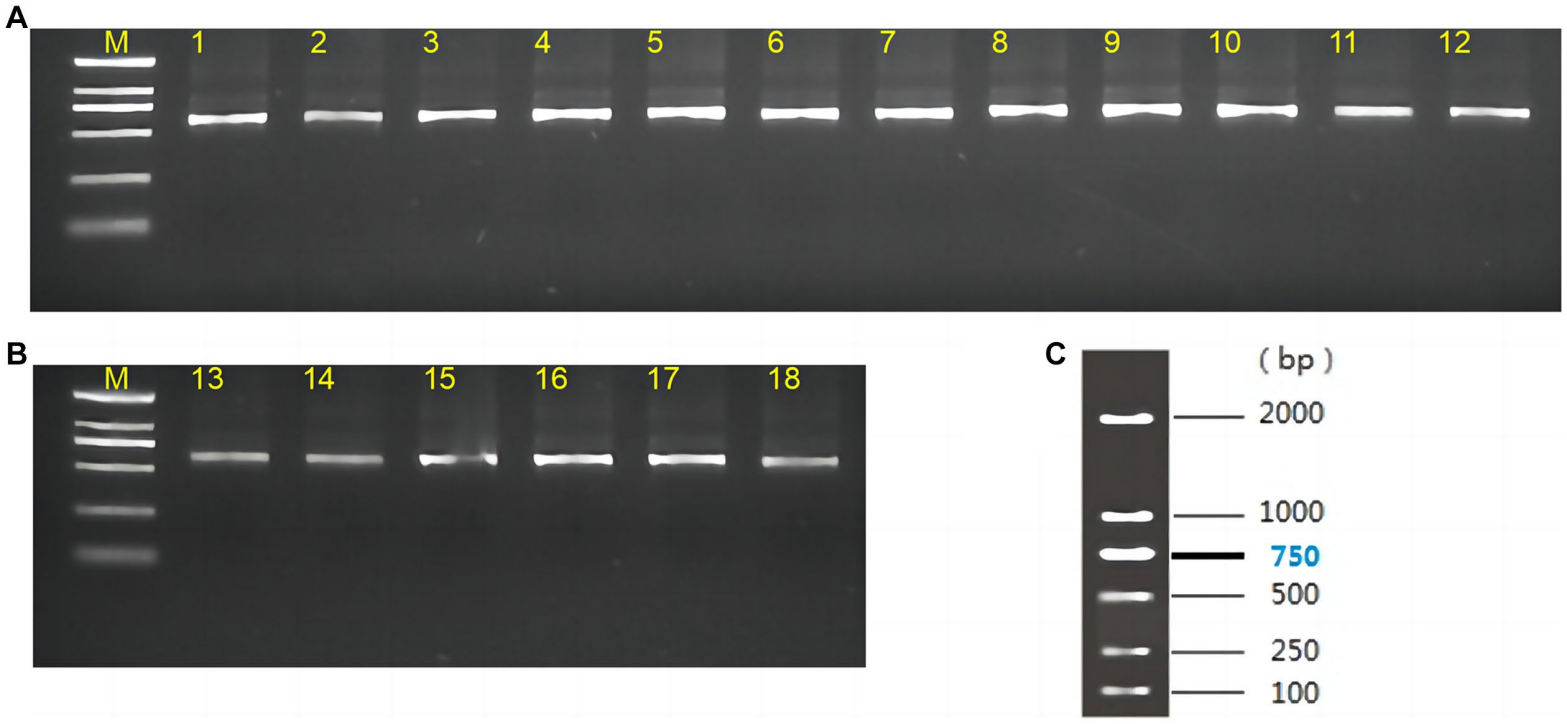
The bands of agarose gel electrophoresis in fecal DNA of the CON group (**A** 1-6), PEO group (**A** 7-12) and CEO group (**B** 13-18) and the distribution of the marker DL2000 bands **(C)**. CON, control group, piglets received basal diet; PEO, piglets received basal diets supplemented with 500 mg/kg plant essential oil (PEO); CEO, piglets received basal diets supplemented with 500 mg/kg coated plant essential oil (CEO).

The results of alpha diversity showed that PEO and CEO supplementation decreased (*p* < 0.05) the Shannon index and increased the Simpson (*p* < 0.05) index in the feces of piglets. The PCoA analysis indicated that the fecal microbial composition in the PEO and CEO group was different from that in the CON group (Figure 5). According to the results of the taxonomy analysis, the community structure composition of different groups at the phylum and genus levels is presented in Figure 6. At the phylum level, Firmicutes, Bacteroidota, Spirochaetota, and Actinobacteriota were the dominant bacteria. The primary bacterial genera were *Clostridium_sensu_stricto_1*, *norank_f__Muribaculaceae*, *UCG-002*, *Terrisporobacter*, and *Lactobacillus*. Dietary PEO and CEO supplementation increased (*p* < 0.05) the relative abundances of *Clostridium_sensu_stricto_1* and decreased (*p* < 0.05) the relative abundance of *Prevotella* in the feces of weaned piglets. The relative abundance of *Terrisporobacter* in the feces of the PEO group was higher (*p* < 0.05) than that in the CON group. The relative abundance of Ruminococcus was increased (*p* < 0.05) in the feces of the CEO group compared with that of the PEO group. The correlation between microbial classification (15 top bacterial genera) and VFA concentrations is shown in Figure 7. The abundance of *Terrisporobacter* was positively (*p* < 0.05) correlated with isovaleric acid concentration. The abundance of *UCG-005* was negatively (*p* < 0.05) correlated with the concentrations of propionic acid, butyric acid, isobutyric acid, and valeric acid. The abundance of the *Prevotellaceae_NK3B31_group* was negatively (*p* < 0.05) correlated with propionic acid and butyric acid. The abundance of *unclassified_f__Lachnospiraceae* was negatively (*p* < 0.05) correlated with valeric acid content. The abundance of *Prevotella* was negatively (*p* < 0.05) correlated with propionic acid, butyric acid, valeric acid, and isovaleric acid. The abundance of *nor-ank_f__Eubacterium_coprostanoligenes_group* was positively (*p* < 0.05) correlated with acetic acid content and the abundance of *Treponema* was negatively (*p* < 0.05) correlated with isobutyric acid. The brands of agarose gel electrophoresis in fecal DNA are shown in Figure 8.

## 4 Discussion

Plant essential oils have attracted increased attention as feed additives in animal nutrition because of their pharmacological and physiological functions (18–20). Growth performance reflects the growth status of pigs, which is directly related to the benefit of pig production. Huang et al. (7) showed that 200 mg/kg PEO supplementation significantly increased ADG and decreased F/G in finishing pigs. Su et al. (9) demonstrated that diets supplemented with 200 mg/kg PEO increased the ADG of weaning piglets. A previous study also showed that piglets from oregano essential oil-supplemented sows exhibited a trend for improved ADG from birth to weaning and reduced the incidence of therapeutic treatment and mortality (10). The possible mechanism is that oregano essential oil can create a physical barrier against microorganisms by inducing a higher glycoconjugate production in the gut, ultimately improving disease resistance and growth performance (21, 22). Piglets supplemented with 0.1% carvacrol-cinnamaldehyde-thymol blend had enhanced body weight at day 14 and increased ADG and decreased FCR between days 1 and 42 (23). A previous research has confirmed that plant essential oil can promote the growth and development of animals by increasing nutrient utilization (24). It has been reported that the coating technique can improve the limitation of high viscosity, pungent odor, and slow release of some PEO, finally increasing the bioavailability of PEO (25). Therefore, ADG in the CEO group was significantly higher and F/G had a decreased tendency compared to that of the PEO group in the current study.

The diarrhea rate of the piglets is related to their resistance to pathogens, which represents the intestinal health status of piglets. Chang et al. (26) showed that a 0.1% mixture of a microencapsulated blend of thymol and carvacrol and phytogenic feed additives increased feed efficiency and decreased the diarrhea of weaned piglets. Research has also demonstrated that 1.5 g/kg red pepper essential oil presented the lowest incidence of diarrhea (27). In this experiment, PEO and CEO supplementation decreased the diarrhea rate of weaned piglets. Essential oil is reported to possess physiological properties to inhibit diarrhea, which may be related to changes in the gut microbiota (28, 29).

Essential oils have excellent antioxidant properties, which can be used to protect the body against oxidant stress (30). The use of PEO as potential feed antioxidants has attracted significant interest and attention since the most common synthetic substances, butylated hydroxy anisole (BHA) and butylated hydroxytoluene (BHT), are suspected to be potentially harmful to the health of animals and humans (31). PEO can exert direct antioxidant effects by impairing the radical chain reaction and achieve indirect antioxidant functions by enhancing the activity of antioxidant enzymes, such as SOD, CAT, and GSH-Px (32). Previous studies have shown that oregano essential oil supplementation increased the activity of SOD and decreased the concentration of MDA in the semen of boar (33). Moreover, MDA concentrations in serum, pancreas, and jejunum were reduced and antioxidant-related gene expression levels of *GPX1* and *SOD1* in the spleen were upregulated by increasing PEO supplementation (8). Pigs fed a PEO diet had higher T-AOC levels than pigs that received a positive control diet (34). Consistent with previous findings, the current study suggests that essential oil could increase the antioxidant capacity of weaned piglets. In addition, CEO supplementation increased CAT activity and decreased MDA content compared to the PEO group. The results might be associated with the increased intestinal dissolution rate of PEO through the cross-linked coating process (16).

Immunity is a physiological function that resists or prevents the infection of microorganisms and harmful substances. Serum concentrations of immunoglobulin and inflammatory factors are important indicators for evaluating the immune function of animals. In this experiment, PEO and CEO addition enhanced the contents of IgA, IgG, and IgM and reduced the contents of IL-1β, IL-6, and TNF-α. A previous study has shown that diets supplemented with different levels of PEO linearly increased IgG and linearly and quadratically increased IgM in weaned pigs (8). Other investigations adding essential oils to the diet of weaner pigs increased IgA and IgM in the blood (35). Dietary 0.025% essential oil supplementation enhanced the concentrations of serum IgA and IgG, finally improving the immunity of weaned pigs (34). However, Li et al. (28) found that diets supplemented with 0.01% of an essential oil product, which contained 18% thymol and cinnamaldehyde, had no significant differences in IgA, IgG, and IgM levels and decreased the concentrations of IL-6 and TNF-α in the plasma of weaned pigs throughout the 5-week feeding trial. The reasons for different results may be related to the composition and dosage of essential oils and the feeding stage of pigs. Piglets fed an essential oil diet tended to have decreased serum TNF-α concentrations compared with those fed a control diet (36). Dietary oregano essential oil supplementation reduced IL-6 mRNA expression and the release of IL-6 in the TNF-α induced IPEC-J2 cells (37). Cheng et al. (38) also found that oregano essential oil (2.5–10 μg/mL) could alleviate inflammatory damage in RAW264.7 cells caused by LPS challenge by inhibiting the expression and secretion of IL-1β, IL-6, and TNF-α. Thymol tended to decrease the production of IL-8 section and significantly downregulated the mRNA abundance of IL-8 and TNF-α in LPS-induced IPEC-J2 cells (39). The results of this study indicate that 500 mg/kg essential oil could enhance the immunity of weaned piglets. Moreover, CEO addition increased IgA concentration and decreased IL-6 concentration compared with the PEO group, indicating that cross-linked coating technology might improve biological availability through the sustained release of PEO in the intestine.

Gut microbiota coexists with the host, which plays an important role in the regulation of metabolic effect, immune function, and intestinal homeostasis (40, 41). The Ace index, Chao index, Sobs index, Shannon index, and Simpson index are used to reflect the alpha diversity of microbial composition. In this study, diets supplemented with PEO and CEO decreased the Shannon index and increased the Simpson index, indicating that the fecal microbial diversity was decreased. Study showed that a mixture of essential oils and medium-chain fatty acids decreased the relative abundance of *Bacteroidetes*, *Bacteroidetes,* and *Enterobacteriaceae* in the feces of weanling pigs challenged with an enterotoxigenic *Escherichia coli* (42). Research has also demonstrated that supplementation with the carvacrol-thymol blend reduced populations of *Enterococcus* genus and *E. coli* in the jejunum (43). Therefore, the decrease in the fecal microbiota diversity after adding essential oil to the pigs may be associated with the inhibition of harmful bacteria.

Study has shown that Firmicutes and Bacteroidetes are the main phyla in the feces of pigs, which promote the absorption of nutrients and energy metabolism of the host (44). Our data revealed that Firmicutes, Bacteroidota, Spirochaetota, and Actinobacteriota were the primary phyla, which is similar to a previous study. *Clostridium_sensu_stricto_1* is a kind of intestinal symbiotic bacteria in the weaning period, which can be colonized in the intestine of newborns within 1 month after birth (45). *Terrisporobacter* can utilize acetic acid and lactic acid to produce butyrate acid under anaerobic conditions, which stabilizes the gut environment (46). *Prevotella* is a newly discovered intestinal microorganism closely related to inflammatory diseases (47). In the current study, diets supplemented with plant essential oil increased the relative abundances of *Clostridium_sensu_stricto_1* and *Terrisporobacter* and decreased the abundance of *Prevotella*, suggesting that plant essential oil could improve fecal microflora by enhancing the abundance of beneficial genera and reducing the abundance of harmful bacterial genera. *Ruminococcus* degrades and converts complex polysaccharides into a variety of nutrients for their hosts. In the current experiment, the abundance of *Ruminococcus* in the feces of the CEO group was increased compared with that of the PEO group. This result indicates that the beneficial bacteria content of feces in the piglets treated with CEO is higher than that of those treated with PEO.

Volatile fatty acids belong to short-chain fatty acids (SCFA), which are produced by gut microbiota during the fermentation of fiber and resistant starch in the gut (48). It is known that PEO has antibacterial and antifungal properties (49). Research has demonstrated that the mixture of essential oils (15% cinnamaldehyde and 5% thymol) enhanced the fecal level of isovaleric acid in weaned piglets (50). Dietary tea tree oil supplementation increased the concentrations of propionate and butyrate in the feces of weaned piglets (51). In this experiment, diets supplemented with CEO increased the concentrations of propionic acid and isovalerate in the feces of weaned piglets, which is consistent with previous studies. The results of correlations between intestinal microbes and VFAs in this study revealed that the abundance of *Terrisporobacter* and *norank_f__Eubacterium_coprostanoligenes_group* was positively correlated with isovaleric acid concentration. The abundances of *UCG-005*, *Prevotellaceae_NK3B31_group*, *unclassified_f__Lachnospiraceae*, *Prevotella,* and *Treponema* were negatively correlated with the VFA concentrations. Similar to our data, Niu et al. (52) found that *Terrisporobacter* was significantly positively correlated with the production of acetic acid, propionic acid, butyric acid, and SCFAs. Liang et al. (53) also showed that *Ruminococ-caceae_UCG-005*, *Prevotella_2*, *Rikenellaceae_RC9_gut_group,* and *Prevotellaceae_UCG-003* were related to the alterations in the fecal metabolites. The results indicated that VFA concentrations were closely correlated with microorganisms in the feces of piglets.

## 5 Conclusion

In the present study, we summarized that dietary PEO and CEO supplementation could improve growth performance, increase antioxidant activity, enhance immune function, and regulate fecal microbiota in the weaned piglets. Moreover, supplemental CEO in the diet has a better promotion effect on growth performance, serum indexes, and fecal bacterial flora than PEO. This study provides a reference for the development and efficient utilization of CEO in weaned piglets.

## Data availability statement

The original contributions presented in the study are publicly available. This data can be found at: https://ncbi.nlm.nih.gov/, PRJNA1046266.

## Ethics statement

The animal study was approved by Animal Ethics Committee of Zhejiang A & F University. The study was conducted in accordance with the local legislation and institutional requirements.

## Author contributions

YN: Conceptualization, Data curation, Investigation, Methodology, Writing – original draft, Writing – review & editing. YC: Data curation, Investigation, Methodology, Writing – review & editing. JL: Methodology, Writing – review & editing. YL: Formal analysis, Software, Writing – review & editing. SX: Formal analysis, Software, Writing – review & editing. CY: Formal analysis, Writing – review & editing. TY: Writing – review & editing, Data curation. WH: Conceptualization, Funding acquisition, Project administration, Supervision, Writing – review & editing.

## Footnotes

^1^http://drive5.com/uparse/
